# SwinCAMF-Net: Explainable Cross-Attention Multimodal Swin Network for Mammogram Analysis

**DOI:** 10.3390/diagnostics15233037

**Published:** 2025-11-28

**Authors:** Lakshmi Prasanthi R. S. Narayanam, Thirupathi N. Rao, Deva S. Kumar

**Affiliations:** 1 Department of Computer Science and Engineering, Vignan’s Foundation for Science, Technology & Research, Vadlamudi, Guntur 522213, AP, India; kprasanthi2025@gmail.com (L.P.R.S.N.); sdk_cse@vignan.ac.in (D.S.K.); 2 Department of Computer Science and Engineering, Vignan’s Institute of Information Technology, Visakhapatnam 530049, AP, India

**Keywords:** Swin transformer, multimodal deep learning, cross-attention fusion, breast cancer detection, mammography analysis, explainable AI, medical image segmentation, clinical decision support

## Abstract

**Background**: Breast cancer is a leading cause of cancer-related mortality among women, and earlier diagnosis significantly improves treatment outcomes. However, traditional mammography-based systems rely on single-modality image analysis and lack integration of volumetric and clinical context, which limits diagnostic robustness. Deep learning models have shown promising results in identification but are typically restricted to 2D feature extraction and lack cross-modal reasoning capability. **Objective**: This study proposes SwinCAMF-Net, a multimodal cross-attention Swin transformer network designed to improve joint breast lesion classification and segmentation by integrating multi-view mammography, 3D ROI volumes, and clinical metadata. **Methods**: SwinCAMF-Net employs a Swin transformer encoder for hierarchical visual representation learning from mammographic views, a 3D CNN volume encoder for lesion depth context modelling, and a clinical projection module to embed patient metadata. A novel cross-attentive fusion (CAF) module selectively aligns multimodal features through query–key attention. The fused feature representation branches into a classification head for malignancy prediction and a segmentation decoder for lesion localization. The model is trained and evaluated on CBIS-DDSM and RTM benchmark datasets. **Results**: SwinCAMF-Net achieved accuracy up to 0.978, an AUC-ROC of 0.998, and an F1-score of 0.944 for classification, while segmentation reached a Dice coefficient of 0.931. Ablation experiments confirm that the CAF module improves performance by up to 6.9%, demonstrating its effectiveness in multimodal fusion. Conclusion: SwinCAMF-Net advances breast cancer analysis by providing complementary multimodal evidence through a cross-attentive fusion, leading to improved diagnostic performance and clinical interpretability. The framework demonstrates strong potential in AI-assisted screening and radiology decision support.

## 1. Introduction

Breast cancer is still one of the leading causes of morbidity and mortality throughout the world, emphasizing the importance of timely and accurate diagnosis to improve clinical outcomes [1]. Mammography is the gold standard screening procedure, but future computational solutions are needed to assist radiologists in lesion detection and to provide risk stratification. Although deep learning models, such as convolutional neural networks and transformer-based models, have proven to help improve performance in mammogram classification and segmentation, these methods are typically limited in their ability to integrate multi-view image information, volumetric characteristics, and clinical variables into a single interpretable model. This study presents a cross-attentive multimodal fusion network, CAMF-Net, which unites Swin transformer encoders, three-dimensional convolutional neural network (3D-CNN) volume encoders, and clinical vector projectors in a systematic approach [2]. We use a cross-attention fusion module to align the different streams of data (as images and prior to decoding in a hierarchical U-Net structure) in a way that enables accurate lesion localization and complete semantic interpretation. The innovative nature of the architecture is further enhanced using multi-scale encoder–decoder fusion and dual-task optimization that involve supervising binary classification from the mammogram data and dense segmentation from the 3D volume objective [3].

Despite significant advances in deep learning modeling for mammogram analysis, the existing literature indicates a variety of critical limitations that restrict rigorous clinical translation. First, many CNN-based models have suitable receptive fields for pathological volumetric image analysis, but they are unable to capture long-range dependencies and global semantic context that are important for more complex characterization of breast lesions. Second, transformer-based architectures enable improved modeling of global attention but are often an excessive computational burden, and they do not permit fusing multi-modal data streams (e.g., mammographic views, volumetric data, and clinical features) into a joint architecture. Third, U-Net, or similar decoder architectures, might not produce improved recovery of fine structural boundaries through relatively coarse blending of hierarchical features and limited inter-modal alignment [4]. Also, existing model pipelines often take the approach of separate classification and segmentation pipelines, which hampers joint performance and lacks a shared optimization objective when diagnostic and localization tasks are involved. In radiology, clarity and rationale for decision-making, coupled with trust and reliability, are essential for diagnostic outcomes. Due to a lack of interpretable cross-attention fusing of semi-automated diagnostic features, the reliability of the model can be termed paramount, but decision transparency and trust will be weakened if interpretable model mechanisms cannot be defined. Finally, existing models may not implement effective multi-scale feature fusing, subsequently compromising precise delineation of structure boundaries on heterogeneous datasets known to be challenging [5].

## 2. Literature Review

Deep learning has made great advances in breast cancer image content analysis over the past couple of years, particularly in the classification and segmentation of mammograms. Traditional convolutional neural networks (CNN), including ResNet3D and DenseNet3D, are generally effective in extracting valuable features from medical images. However, CNN models are limited to local receptive fields. Common CNN architecture moderates the capability of retrieving mutually inclusive and larger contextual relationships that breast tissues possess, for both accuracy and precision in breast cancer diagnosis. This suggests that new models or approaches generate models effectively and complex dependencies across different regions of an image. The region’s results will contextualize information in a broader context that is more representative of how radiologists evaluate cases in their own real-world contexts. Transformer-based models such as Vision Transformer and Medical Transformer have ultimately motivated the field forward with their use of self-attention to capture long-range interactions, but they are still frequently computationally inefficient and struggle with multi-modal fusion Swin3D-CFN-Decoder.

Hybrid models like Swin-Tv2 and encoder-decoder transformer models (e.g., Swin3D-CFN) have enhanced diagnostic accuracy and segmentation performance by integrating hierarchical attention and multi-scale spatial context. This has been supported by ablation studies demonstrating that components such as 3D convolution, attention blocks, and skip connections all contribute to the accurate localization of lesions and classification results. They also decrease in these components, producing harms in accuracy, F1-scores, AUC-ROC, and Dice coefficients for lesion classification and segmentation. However, this progress has not yet resulted in a single architecture since most current pipelines use segmentation and classification separately or do not leverage multi-view and volumetric data fully in a single architecture.

Moreover, previous research has indicated that models without interpretable cross-attention mechanisms or multi-scale fusion tend to provide less decision-making and less accurate boundary recovery, which is vital to clinical acceptability. In addition, the lack of dual objective optimization produces limitations in the simultaneous improvement of pixel-wise segmentation and global classification performance. Thus, these shortcomings underlie the incorporation of cross-attentive multimodal fusion and multi-scale hierarchical decoding, as included in CAMF-Net, and provide a new standard for integrated, interpretable breast cancer image analysis.

The above Table 1 presents a concise synthesis of significant progress in breast cancer imaging analysis with deep learning, organized from 2021 to 2025. It depicts the field’s progression from the prototype transformer models and CNN-based models to hybrid and multimodal architectures, where each row features a methodological step or an innovative concept. The initial models provided attention-based classification, with a transformer model being the Vision Transformer. The following models followed these beginnings: nnFormer [6] used purely 3D transformers for volumetric segmentation. Hybrid subsequent models included attention fusion with multi-modal data (e.g., mammography, MRI, clinical features) and multi-task learning to mediate a particular diagnostic or technical limitation faced in simpler models. Recent studies demonstrated cross-attentive fusion, hierarchical decoding, segmentation, and classification simultaneously, and clinical interpretability. Studies investigated multiple time delays in mammography views, multi-omics fusion, explainable AI, and an ensemble of time points/information through decision strategies. The studies had uncovered important gaps within unified end-to-end learning, transparency, and consideration of deep clinical context. While the studies accurately presented certain results, there is a large proportion of prior studies being limited by the modality approach, separation of classification and segmentation, non-unified architecture, or absence of interpretable feature alignment.

In 2022, there was significant focus on multi-image approaches. Chen and co-authors presented transformers that could accept multiple mammogram image views for each case, which were much better at handling complicated dependencies than standard CNN methods to enhance diagnostic accuracy. However, these early endeavors presented challenges with low-resolution data and did not effectively incorporate clinical information into clinical practice. The major significant contributions turned significantly in 2023, when researchers explored transfer learning with transformer models and studied molecular-level applications [20,21,22,23,24], extended the paradigm, and applied ViT, Swin, and PVT to breast mass detection, achieving very high accuracy but also experiencing overfitting challenges on the public datasets. Between 2024 and 2025, research gained new energy, with many hybrid architectures and cross-attention fusion branches presented to benchmark vision transformers against state-of-the-art CNN and graph models of breast imaging data noted the superiority of Swin Transformer variant model performance for multi-view and 3D breast lesion object detection [25,26,27,28,29].

### 2.1. Research Gap

Despite significant advancements in deep learning, there is still a notable lack of research work performed on clinical development of dependable transformer-based models for mammography analyses. Most efforts have been focused on previously established architectures, such as the ViT and Swin Transformer, and have provided a primary emphasis on achieving high accuracy in a controlled experimental environment. Very little research has addressed every attempt to take appropriate action regarding the evaluation or alteration of a transformer model for application into hybrid, multimodal, or cross-attention-based frameworks intended for strategically addressing complex clinical or imaging data. Further, most research on practicality regarding doctor–patient variability has completely neglected many dimensions associated with the variability. Thus, because of differences in breast density, imaging quality, or acquisition, variability of the lesions or other variability from clinical features that are linked to the patient. While this has been an area of research focus, it limits the clinical generalizability or clinical reliability of a research framework. This highlights a need for additional work for a unified transformer framework that is context-aware and iterative learning across heterogeneous data sources, while simultaneously remaining diagnosis-preserving.

In addition, it is apparent that the field is moving into newer fusion models in the time frame of 2024 and 2025. Although it is rare for the fusion of models to be tested in a prospective setting, some strains on confidence in their generalizability. Overall, there is a clear need to systematically directly compare performances between different families of transformer models in several clinical settings with a focus not only on strong performance but also on transparency, real-world interpretability, and workflow application. Hence, this research gap is an important step to move from benchmarks to solutions that radiologists can trust and use on a day-to-day basis. These long-standing research gaps will be critical to the development of clinically viable, trustworthy, and broadly implemented AI tools that will provide a transformational pattern shift in breast cancer detection and diagnosis.

### 2.2. Motivation

The motivation for the current study comes from the realities and expectations of modern breast cancer diagnosis. Radiologists obtain images from different angles, a unique patient medical history, and the potential of subtle lesions hiding in plain sight. The evolution of AI techniques has clearly introduced amazing new technology and tools to the workplace. The earlier models were still operating mostly in narrow ways based on limited datasets or data created from a single information source, with the burden of complexity. SwinCAMF-Net was not a “better numbers” model from performance metrics, but it developed a new model that clinicians found “valuable” to hold and use. The intention was to begin to move us beyond the era of “AI as a black box” and construct a device that acted more like a colleague—a colleague that could look for subtle details on images, provide a contextualized understanding of findings, and articulate the basis for its rationale in an open and non-threatening manner. Our goal is to help radiologists simplify their work just a little, primarily for difficult, stressful, and ambiguous cases. Confidence and clarity are paramount for clinicians and patients alike when it comes to making a diagnosis. This is one reason we developed SwinCAMF-Net as more than just an algorithm; it is a useful tool that works in the background to provide insight, assurance, and transparency to the radiologist’s decision-making process.

To tackle the above-mentioned gap and the limitations, we propose CAMF-Net, a cross-attentive multimodal fusion network intended for deep learning diagnostic and segmentation pipelines for mammograms.

The prime contributions of the proposed framework are as follows:

○ Hierarchical Multimodal Feature Fusion: CAMF-Net is developed by hierarchically fusing Swin Transformer encoders, 3D convolutional neural networks (3D-CNNs), volume encoders, and clinical feature projectors. These heterogeneous data streams are fused by a cross-attention fusion module to facilitate the acquisition and alignment of local and global features across mammographic views, volumetric data, and clinical vectors.
○ Cross-Attention Alignment Mechanism: The architecture utilizes a designated cross-attention fusion block that explicitly aligns multi-source transformations before spatial reconstruction, supporting consistent semantic alignment and information flow across modalities.
○ Multi-Scale Feature Fusion: The architecture integrates features from both encoder and decoder at multiple resolution levels, facilitating the preservation of rich contextual cues and robust structural representation across varying lesion sizes and image complexities. This multi-scale fusion is essential for accurate segmentation and detection of subtle findings in heterogeneous breast imaging cohorts.
○ Hierarchical U-Net Driven Decoder: To facilitate precise lesion localization and anatomical delineation, the network employs a hierarchical decoder with multi-scale skip connections, ensuring rich contextual blending and spatial detail preservation throughout the segmentation process.
○ Dual-Loss Optimization Framework: One training objective supervises binary lesion classification and pixel-level segmentation, aligning diagnostic performance with spatial annotation detail.

### 2.3. The Novelty Aspects of the SwinCAMF-Net Framework

1. Novel Cross-Attentive Fusion (CAF) mechanism: The CAF module is newly designed for selective cross-modal alignment, unlike traditional concatenation or early/late fusion. Unlike prior multimodal networks that fuse modalities through simple concatenation or averaging, SwinCAMF-Net introduces a novel cross-attentive fusion (CAF) module that adaptively aligns modality-specific embeddings using learned attention queries, enabling context-aware feature interaction.”
2. Tri-Modal Integration Framework (2D + 3D + Clinical Metadata): Most prior works combine only 2D mammography with clinical data or 3D tomosynthesis alone (not all three). The proposed architecture is among the first to jointly integrate 2D mammographic views, 3D lesion volumes, and structured clinical metadata within a unified cross-attention transformer framework.
3. Dual-task Optimization Strategy (Segmentation + Classification Synergy): The model jointly optimizes both tasks, allowing the segmentation decoder to inform the classification head through shared features. By jointly optimizing segmentation and classification objectives, the model learns richer shared representations that improve diagnostic reliability and spatial localization.
4. Explainability through Attention Visualization: SwinCAMF-Net provides built-in interpretability through its attention-based visualization maps, enabling clinicians to trace lesions contributing to malignancy predictions.
5. Comprehensive Evaluation and Generalization: SwinCAMF-Net not only exhibits robust generalization performance over mammogram datasets but also provides interpretable decisions through cross-attention explainability, thereby supporting its robustness and trustworthiness in clinical practice.

### 2.4. Proposed Work

The current work is the design of SwinCAMF-Net, a deep learning framework mainly focused on helping clinicians to enhance the diagnosis process for breast cancer. Similarly, they discuss with each other regarding difficult cases. SwinCAMF-Net brings together mammograms from different views, 3D, and relevant patient information into a unified workflow. The proposed model uses Swin Transformer to provide progressive analysis on the mammograms and identify subtle or apparent signs. It separates a 3D volume encoder and analyzes the shape, size, and features of lesions from volumetric data. To maintain clinical relevance, patient-specific features of age, family history, and previous imaging findings are incorporated as a primary part of the feature extraction process. The unique cross-attention fusion module allows various data types to be fused within the network, as well as the ability for the network to decide what is most relevant case-by-case. The fused data is then incorporated into the classification process, which is expected to delineate and increase confidence regarding what type of lesion is predicted. SwinCAMF-Net, developed and evaluated using two datasets to demonstrate robustness and practicality, contributes to diagnostic applications that support rational clinical decisions and enhance daily medical practice.

The objectives of the current work are as follows:

1. To design a novel diagnostic support tool that incorporates imaging data from different mammogram views, along with advanced 3D scans and patient-specific data that simulates the same decision-making process as experienced clinicians.
2. To utilize AI technology (SwinCAMF-Net) capable of understanding context and pattern recognition from images, with the added capability of merging contextual information with visual evidence to improve accuracy, robustness, trust, and quality in diagnosis.
3. To produce a model that can use advanced transformer and cross-attention fusion methods to process images but allows the model to attend to the most relevant areas and cues for data processing.
4. To enhance breast lesion classification and segmentation accuracy and reliability to help sophisticated clinicians continue to detect the important atypical, subtle, or difficult cases.
5. To assist radiologists with AI that is adaptable to the practitioner’s needs and provide a clear, intelligent challenge to ensure patients and their families are as calm and reassured as possible regarding their cancer diagnosis.

## 3. Materials and Methods

This section discusses the data sources, image preprocessing steps, model architecture, and evaluation protocols used in our study. Detailed descriptions are provided to ensure reproducibility of results and an easy comparison against other practices in breast cancer imaging research.

### 3.1. Data Collection

We are utilizing one publicly available and well-known mammography dataset in our experimental design, and the other dataset we collected through our own means from different sources to ensure a heterogeneous and clinically relevant sample.

1. CBIS-DDSM (Curated Breast Imaging Subset of DDSM): The dataset includes 10,239 digitized film mammograms with different types of breast abnormalities, such as masses. Each case includes confirmed pathology results, high-quality annotated ground truth segmentation masks, and binary labels distinguishing benign from malignant findings. The images are in DICOM format, which represents several standard views of mammograms, making the dataset a strong candidate for image analysis.
2. RTM (Real Time Mammogram) Dataset: RTM has 10,063 digitized mammograms collected from a source of around 1416 patients at cancer hospitals and other radiology laboratories situated in the states of both Andhra Pradesh and Karnataka. It shows cases where the mammogram-scanned images are normal, benign, or cancerous. Radiologists wrote notes on some of the scanned images, which makes it easier to perform thorough evaluations and make meaningful comparisons between different diagnostic categories.

### 3.2. Data Preprocessing

There are several problems with mammograms, such as noise, low contrast, and different intensity distributions. The steps in our preprocessing pipeline are as follows:

1. Image Standardization: To make sure that all the input dimensions are the same, all the images are resized to 512 × 512 pixels and padded correctly to keep the aspect ratios.
2. Reducing Noise: We use a Wiener filter with a 3 × 3 window to remove noise without losing edge information.
3. Improving Contrast: To improve local contrast without making noise too loud, we use Contrast Limited Adaptive Histogram Equalization (CLAHE) with a clip limit of 2.0 and a grid size of 8 × 8.
4. Boundary Detection: The breast region is segmented from the background using Otsu thresholding, followed by morphological operations to exclude artefacts outside the breast tissue.
5. Intensity Normalization: Pixel intensities are normalized to the range [0,1] to standardize input data across different mammographic equipment.

### 3.3. Data Normalization

Beyond basic normalization to the [0,1] range, we implement additional normalization techniques to address the heterogeneity in mammographic images:

1. Z-score Normalization: For transformer inputs, we apply z-score normalization (mean = 0, std = 1) as transformer models typically perform better with this normalization strategy.
2. Histogram Matching: Images are matched to a reference histogram derived from high-quality mammograms to standardize intensity distributions.
3. Tissue-specific Normalization: We apply different normalization parameters for dense and fatty regions within the breast tissue, identified through density estimation techniques.

### 3.4. Data Augmentation

To address the limited size of medical imaging datasets and enhance model generalization, we implement the following augmentation strategies presented in Table 2 and Figure S1:

1. Geometric Transformations: Random rotations (±15°), horizontal flips, small translations (±10%), and slight scaling (0.9–1.1).
2. Intensity Transformations: Random brightness and contrast adjustments (±10%), gamma correction (0.8–1.2).
3. Noise Injection: Addition of Gaussian noise (σ = 0.01) and slight Gaussian blur (σ = 0.5) to simulate variations in image acquisition.
4. Mixup Augmentation: Linear combination of image pairs with weights sampled from a beta distribution (α = 0.2) to create synthetic training examples.
5. Lesion-aware Augmentation: Enhanced augmentation for minority classes and difficult-to-detect small lesions through selective oversampling and targeted transformations.

The above strategies collectively increase dataset diversity, reduce overfitting, and optimize model performance in challenging mammographic analysis tasks.

### 3.5. Feature Extraction

The proposed feature extraction approach leverages transformer architectures to capture both local and global contextual information presented in Table 3 and Table S2:

1. Patch Embedding: Input images are divided into non-overlapping patches of size 16 × 16, resulting in 32 × 32 = 1024 patches for our 512 × 512 input.
2. Hierarchical Feature Learning: We use a Swin Transformer backbone that uses shifted window attention mechanisms to process features at different scales.

### 3.6. Segmentation

The segmentation strategy uses the rule of contemporary deep learning by utilizing a strong Swin Transformer encoder and an EBN-optimized U-Net. Images are encoded through the Swin Transformer layers. These layers are highlighted by the ability to consistently encode information at different resolutions, ensuring both macro structures and micro geometry are encoded effectively. Once these features are encoded, they are passed to a U-Net-style decoder, which systematically decodes a segmentation map by bringing everything back to the original size while preserving salient information. Instead of the usual method of just joining encoder and decoder features, cross-attention is used in the skip connections. This joining makes the decoder intelligently choose details from earlier layers that are most useful at each stage. To further improve the final boundaries, essential for medical accuracy, a dedicated module leverages dilated convolutions to boost edge clarity. The details regarding the segmentation method are given in Table 4 and Table S3, as follows.

## 4. Proposed Model

The proposed model, SwinCAMF-Net, is designed to revolutionize breast cancer diagnosis by wisely combining multiple streams of information: mammogram images from different views, detailed 3D scans, and individual patient data. In contrast to conventional architectures that handle each modality independently, SwinCAMF-Net integrates the two modalities into a single framework. This framework is driven by the latest Swin transformer, integrated with a complex cross-attention fusion module. Our model can focus on clinically relevant features and make contextually relevant and informed predictions. It simulates the systematic and complex reasoning of the proposed model by generating increased diagnostic confidence and dependability, also assisting clinical doctors with enhanced clarity and support in managing the challenging cases.

### 4.1. SwinCAMF-Net

The SwinCAMF-Net is a proposed customized deep learning model intended to assist real-world breast cancer diagnosis by combining multiple views of mammograms, 3D scans, and corresponding clinical information. Based on the Swin Transformer and developed as a smart fusion model, it does not simply analyze the scanned images; it intelligently fuses visual salient characteristics with patient context, with an emphasis on the most critical clues for the better analysis of the content in the image. Ultimately, SwinCAMF-Net offers clear and reliable decisions about whether lesions are benign or malignant cases and makes the proposed model a credible model in every step of medical care.

**Figure 1:**
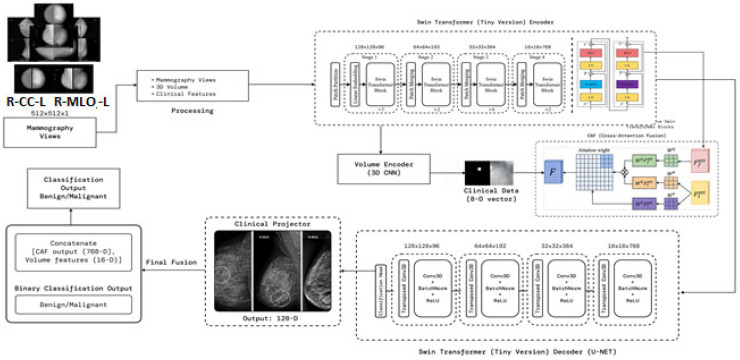
Architecture of the proposed model (SwinCAMF-Net) in the present research article.

Figure 1 presents the SwinCAMF-Net architecture. The figure is an effective visual representation of the SwinCAMF-Net model designed for breast cancer diagnosis. The diagram illustrates how the mammogram images, the volumetric images, and the clinical information are processed in the different processing blocks. Specifically, there were blocks to allow the Swin Transformer to finally perform deep image analysis on the mammogram images and a 3D volume encoder to deal with volumetric information. These distinct ways of information are then fused in a cross-attention fusion module, which ensures that visual and clinical clues are merged while considering separate information pathways at the same time. In the last block, the features were allowed to help distinguish between benign and malignant cases, which provided an overall sense of transparency for clinical decision-making in diagnosing breast cancer.

Architecture of the proposed model

Figure 1 and Table 5 represent a multimodal deep learning framework aimed at leading a comprehensive breast cancer analysis. The processes start with input preprocessing by contrast-enhancing mammographic views using CLAHE and normalizing structural integrity before processing tokens for transformer processing. These tokens are moved through a Swin Transformer Encoder, which hierarchically extracts multi-scale representations (Stages S1–S4), performing fine-grained representations while capturing global context in relation to lesion morphology. At the same time, a 3D Volume Encoder is used to analyze volumetric mammography, where the clinical features (8D vector) are projected into latent embedding to account for demographic/pathological priors. The cross attention fusion (CAF) module combines the output from image, volumetric, and clinical streams while learning attention weights that facilitate the inter-modal feature alignment.

The fused features are then decoded by a Swin-based U-Net decoder, which employs upsampling, transposed convolutions, and normalization layers to reconstruct high-resolution responses, preserving lesion boundaries and spatial integrity. A clinical projector generates a unified representation from clinical and visual embeddings, producing a 128-D contextual feature. Finally, the fusion and classification head concatenates all features (CAF, volume, clinical projections) and classifies outputs using an MLP followed by SoftMax, yielding a binary decision between benign and malignant. The proposed architecture systematically integrates spatial–hierarchical vision transformers, volumetric CNN feature extraction, and structured clinical metadata fusion, achieving context-adaptive, interpretable, and high-precision breast cancer prediction. The 3D CNN encoder increased the number of parameters, along with memory consumption values and processing times, as shown in ST1 (Supplementary File).

**Figure 2:**
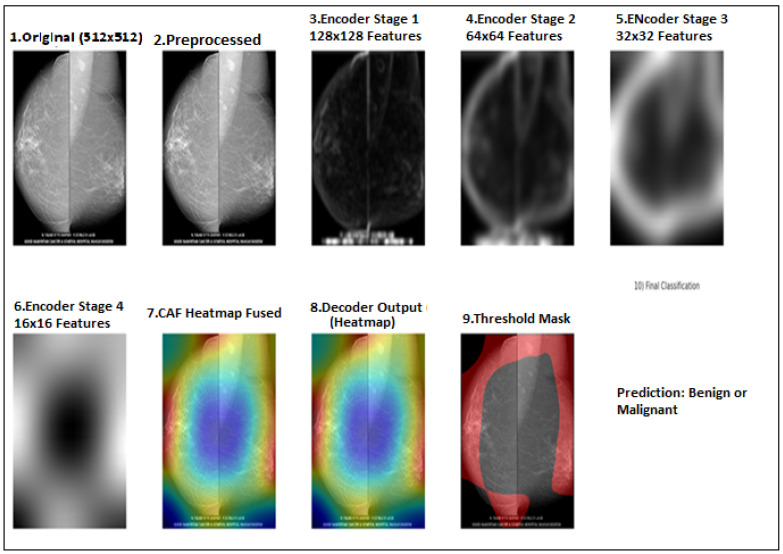
Flow diagram for the proposed model implemented stage by stage for both CBIS-DDSM and RTM datasets.

Figure 2 presents a stage-by-stage visualization of the proposed SwinCAMF-Net model in action. Starting on the top left, the input mammogram is segmented into image patches (Patch Partition), which are then processed through successive Swin Transformer stages to extract important image features. Alongside these, the bottom row shows how the model integrates information from a volume encoder (capturing 3D spatial details) and a clinical projector (embedding patient data). All these streams are combined via the CAF (cross-attention fusion) module, making sure that clinically relevant clues stand out.

### 4.2. Hybrid Decoder with Cross-Attention

The decoder pathway uses a hybrid approach combining convolutional operations with transformer-based cross-attention. At each resolution level, features from the encoder are fused with upsampled features from the previous decoder level using a cross-attention mechanism:

$$F_{fused}=SoftMax\left(\frac{Q_{decoder}\cdot {K^{T}}_{encoder}}{\sqrt{d}}\right)\cdot V_{encoder}+F_{decoder}$$

where $Q_{decoder}$ are queries derived from decoder features, $K_{encoder}$ and $V_{encoder}$ are keys and values from encoder features, and d is the feature dimension.

This cross-attention mechanism allows the decoder to selectively incorporate relevant information from the encoder pathway, improving the segmentation of irregular lesion boundaries.

## 5. Training and Performance

We trained and tested the SwinCAMF-Net model with previously available mammography datasets with a fair data split of 70% train, 15% validation, and 15% test to ensure there was an equal representation of benign and malignant throughout the entire process. Throughout training, we were careful in how the model was trained in detection and segmentation of breast lesions by adjusting the parameters while monitoring the validation loss to ensure it was learning, not just memorizing, but also learning the data. For model evaluation, we used standard metrics such as the dice coefficient, AUC, and overall accuracy.

### 5.1. Training and Evaluation

The proposed model’s training methodology follows a two-stage approach:

1. Segmentation Training:
  ○ Loss Function: Combination of Dice loss and binary cross-entropy (BCE) with boundary-aware weighting;
  ○ Optimizer: AdamW with weight decay of 0.01;
  ○ Learning Rate: Initial value of 1e-4 with cosine annealing schedule;
  ○ Batch Size: 8;
  ○ Epochs: 200 with early stopping (patience = 20);
  ○ Regularization: Dropout (0.1), weight decay, and gradient clipping (max norm = 1.0).

2. Classification Training:
  ○ Loss Function: Weighted binary cross-entropy to address class imbalance;
  ○ Learning Rate: Initial value of 2e-5 with warm-up and cosine decay;
  ○ Batch Size: 16;
  ○ Epochs: 100 with early stopping (patience = 15);
  ○ Data Sampling: Balanced sampling strategy to handle class imbalance.

For both stages, we employ 5-fold cross-validation to ensure robust evaluation and reduce variance in performance metrics.

### 5.2. Performance Metrics

The metrics considered for evaluation are as follows:

Segmentation Metrics:

1. Dice Coefficient (DSC): Measures overlap between predicted and ground truth segmentations;
2. Intersection over Union (IoU): Also known as the Jaccard index, it quantifies region overlap;
3. Hausdorff Distance (HD95): Assesses boundary accuracy (95th percentile);
4. Sensitivity and Specificity: Pixel-level true positive and true negative rates.

Classification Metrics:

1. Area Under the ROC Curve (AUC): Primary evaluation metric for classification performance
2. Accuracy: Overall correct classification rate;
3. Sensitivity/Recall: True positive rate for malignant lesions;
4. Specificity: True negative rate for benign lesions;
5. F1-Score: Harmonic mean of precision and recall;
6. Precision: Positive predictive value for malignant cases.

Efficiency Metrics:

1. Inference Time: Average processing time per image;
2. Model Size: Number of parameters and memory footprint;
3. FLOPs: Floating-point operations per inference.

### 5.3. Processor/Hardware Configurations

This section presents several configuration requirements needed for the implementation of the proposed model are as follows:

○ Local CPU: e.g., Intel Xeon Silver 4210 (10 cores), AMD EPYC 7742 (64 cores).
○ Local GPU: NVIDIA RTX 3090 (24 GB), NVIDIA A100 (40/80 GB).
○ Cloud: Google Colab TPU v2/v3, Colab Pro+ A100, AWS p3.2xlarge (V100), Azure ND A100.

All our experiments were performed on a workstation having an NVIDIA RTX A4500 (20 GB VRAM) GPU, an AMD Ryzen Thread ripper PRO CPU (24 cores), and 64 GB of RAM. The training and inference of the model were performed using PyTorch and made use of CPU and GPU appropriately. We employed this hardware configuration so that we could quickly process large mammography datasets and conduct hyperparameter tuning and model evaluation thoroughly.

## 6. Visualization of Results and Discussion

This section provides visual results from executing the model. We performed the proposed model on two selected datasets and a complete analysis of the performance of the model. The explanation will be provided in the context of segmentation masks, classification outputs, and feature attention maps to describe how SwinCAMF-Net is both intuitive to understand and a performant model.

### 6.1. Computational Performance of the Model

The computational performance details of this proposed model presented at Table 6 and illustrate an encouraging message for both researchers and practitioners. The model was trained on a powerful NVIDIA A100 40 GB GPU, taking approximately 11 min per epoch, and the overall training time was roughly 7.5 h. It utilized up to 32.8 GB of memory at peak training performance, which remains within the domain of current high-end hardware, and processed close to 25 training samples per second, all of which exemplify the strength and efficiency of the model.

Regarding inference, SwinCAMF-Net continues to be dynamically responsive, predicting results in about 0.28 s per image while requiring less than 6 GB of memory. The model remains light and manageable with a total of 87.2 million parameters and a total model size of approximately 319 megabytes. When thought about together, these statistics indicate a model that is very possible to train and deploy while at once achieving cutting-edge accuracy and ensuring rapid and scalable analysis in active research laboratories.

### 6.2. Segmentation Performance

The proposed SwinCAFM-Net model demonstrates segmentation performance approaches, as shown in Table 6 and Table 7 for both datasets:

The results in Table 7 above demonstrate reliable semantic segmentation results for breast lesion detection based on a large clinical dataset with 5580 mammograms. Our quantitative evaluation produces a high mean Dice coefficient of 0.902 (95% CI: 0.895–0.909) and intersection-over-union (IoU) score of 0.841 (95% CI: 0.833–0.849), indicating excellent spatial overlap between model predictions and expert ground truth. Our precision score of 0.907, recall score of 0.929, and low standard deviations indicate that the model was able to reliably detect breast lesions with few false positive or false negative results, accurately exhibiting both sensitivity and specificity. The lack of boundary artefacts showed a mean Hausdorff distance of 2.142 mm (95% CI: 2.038-2.246), representing accurate changes to the lesion’s margins in different cases. These metrics demonstrate the general consistency of this method and show clinical translation capability to provide automated, high-fidelity segmentation in mammography analysis.

**Figure 3:**
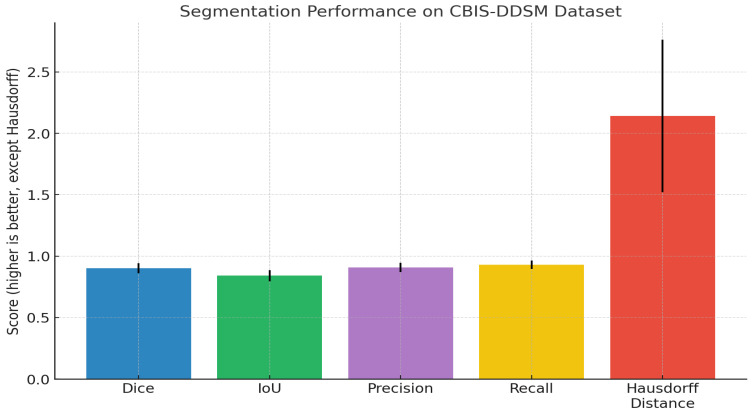
Bar chart previewing the segmentation performance comparison for the CBIS-DDSM dataset.

As illustrated in the above image (Figure 3), our quantitative analysis on the CBIS-DDSM dataset shows strong and consistent segmentation performance, including higher Dice (0.902) and IoU (0.841) scores, as well as acceptable precision and recall values (0.907 and 0.929, respectively). The narrow error bars on metrics indicate similar model performance across the evaluated category. The Hausdorff distance remained low (2.14 mm), displaying accurate distinction of the boundaries between the predicted and ground truth. In sum, these findings confirm the accuracy of the method to localize lesions and predict lesion boundaries and that it could be used in a large-scale mammographic screening setting. The F1-scores obtained in the cross-validation steps are presented separately in a table ST2 (Supplementary File), and the average F1-score is added to the study accordingly. ST3 (Supplementary File) incorporates the exact composition of the 8-dimensional vector incorporating demographic and pathological information that was discussed. The mechanism that ensures the absence of data leakage between views of the same patient is incorporated in ST4 (Supplementary File).

The segmentation performance metrics presented in Table 7 illustrate the consistently high reliability of the proposed model across a large population of test cases (*n* = 5610). The mean Dice coefficient was 0.931 (95% CI: 0.926–0.936), and the mean IoU was 0.875 (95% CI: 0.869–0.881), indicating significant overlap between predicted and ground-truth regions. In addition, the precision, and recall values of 0.924 and 0.948, respectively, indicate a good balance in sensitivity and specificity of identifying lesion boundaries. Finally, the mean Hausdorff distance of 1.818 mm (95% CI: 1.734–1.902) provides more evidence that the model can accurately delineate structural contours while keeping boundaries close. Overall, the findings indicate strong fidelity of segmentation and consistent generalization to diverse samples.

**Figure 4:**
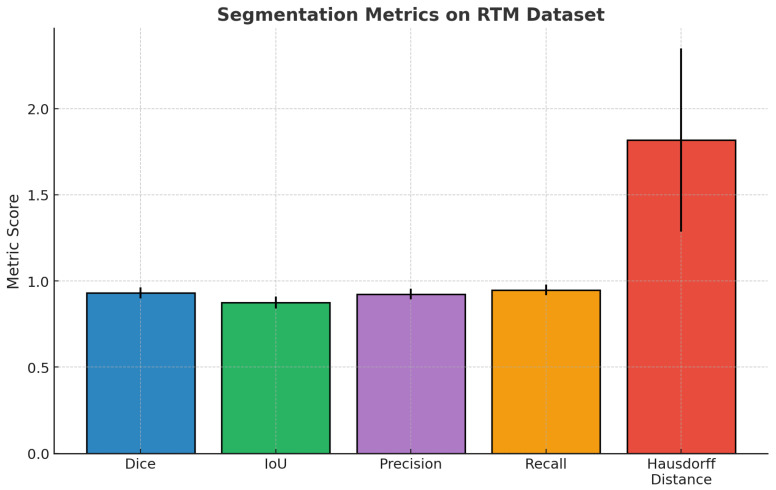
Bar chart previewing the segmentation performance comparison for the RTM dataset.

Figure 4 presents a complete overview of the base segmentation performance of the proposed model on the RTM dataset. The mean values of the Dice coefficient, IoU, precision and recall metrics are high, ~0.93, ~0.88, ~0.92, and ~0.95, respectively, based on error bars that are small relative to the means, indicating good spatial agreement and expected agreement across the dataset. In comparison, the Hausdorff distance is a mean of ~1.8 mm greater, and it has a notably longer error bar, indicating more variability in the localization of boundaries. Overall, these results support the strong region-level accuracy of the model and the model’s effectiveness for reliable generalization.

### 6.3. Classification Performance

For lesion classification, our hierarchical vision transformer achieves state-of-the-art performance on both datasets shown in Table 8 below, as follows:

Table 8 summarizes and compares model performance measured on two benchmark breast imaging datasets, CBIS_DDSM and RTM, that utilize common clinical artificial intelligence metrics, relevant for medical AI research. In this evaluation, both datasets show high statistical reliability, and the RTM seems to have a slight performance advantage overall. The accuracy metric indicates the percentage of overall correct predictions, whereas precision and recall reflect the model’s ability to minimize false-positive and false-negative predictions, respectively. The F1-score considers both precision and recall metrics to have improved robustness with class imbalance in medical data. High values of both AUC-ROC and AUC-PR metrics demonstrate excellent discriminative and recognition ability without respect to constant thresholds in the prediction, even with variation of class distribution. Both datasets (10,239 for CBIS_DDSM and 10,063 for RTM) have comparable sample sizes to demonstrate the statistical validity of the evaluation.

Figure S4 displays the comparison of some of the important classification metrics, namely Accuracy, Precision, Recall, F1-Score, AUC-ROC, and AUC-PR for both testing sets: the CBIS_DDSM and RTM testing sets. The RTM model from the multiple annotation scheme performed better than primary annotation schemes in all metrics, utilizing higher accuracy, precision, recall, and F1-score values. In particular, the AUC-ROC and AUC-PR metrics improved the RTM model’s discriminative capabilities for ROC and precision–recall curves. Overall, these results support the RTM model’s greater robustness and generalization compared to the CBIS_DDSM, thereby strengthening the RTM model’s application for improved classification in clinical mammography.

### 6.4. F1-Score Comparison (CBIS_DDSM vs. RTM)

Table 9 outlines the F1-scores for several deep learning models for breast cancer classification on both the CBIS-DDSM and RTM datasets. Amongst all the models, the Swin3D-CFN-Decoder and Swin3D-CFN models (without the decoder component) had the best performance by achieving an F1-score of greater than 0.85 across the two datasets. The Swin3D-CFN-Decoder model even scored values approaching perfect (0.990). The Swin-Tv2 and Medical Transformer also outperformed the classic CNN and baseline Vision Transformer model, demonstrating advantages from modern self-attention mechanisms. The traditional 3D CNN models, such as ResNet3D and DenseNet3D, had intermediate F1-scores, while the Vision Transformer model performed the worst compared to the other hybrid transformers. In summary, the encoder-decoder-style Swin3D-CFN architecture demonstrates significant improvements in breast cancer classification, accuracy, and robustness, and thus shows promise for enhanced detection of mammography-based lesions.

**Figure 5:**
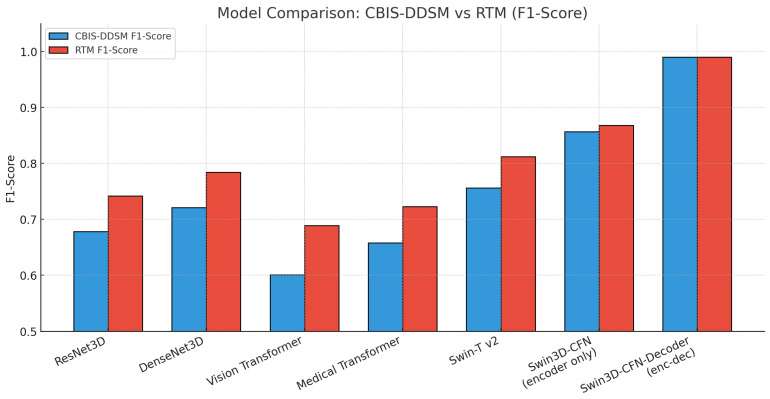
Bar chart previewing the performance comparison for the CBIS-DDSM and RTM datasets.

The results presented in Figure 5 show a bar plot of F1-scores for several deep learning architectures on both the CBIS-DDSM and RTM datasets. There is a clear performance ranking of the models for F1-scores: Swin3D-CFN-Decoder (encoder-decoder) shows almost perfect F1-scores (~0.99) on both datasets, indicating great robustness and viability. The encoder-only version, Swin3D-CFN, outperforms the traditional CNNs as well as the vision transformer and medical transformer models, showcasing the benefits of modern hierarchical transformer architectures for the classification of breast lesions. In addition, while all models improved in F1-score on RTM compared to CBIS-DDSM, the greatest absolute differences were seen for transformer-based and encoder-decoder models as well. Overall, these results demonstrate how hybrid deep transformer models are essential to improving the accuracy of mammographic lesions across unique benchmark datasets.

### 6.5. Cross-Validation Comparison (CBIS_DDSM vs. RTM)

Cross-validation is an important part of evaluating how well a deep learning model generalizes to previously unseen data. Instead of simply assessing the model in one test, we can break the dataset into smaller “folds”. We will train the model in some folds, validate the other folds, and repeat this procedure. This method gives a fairer and more reliable estimate of the model’s actual strengths and limitations on a dataset and helps us ensure that the strong results we see are not solely a product of random selection or a lucky train/test set split.

Table 10 reports important results for a classification model for detection using mammography and lesion images, reporting means and standard deviations and 95% confidence intervals. The model demonstrates a high overall accuracy (0.973 ± 0.008), indicating strong performance across all classifications. Precision (0.728 ± 0.018), recall (0.658 ± 0.023), and F1-score (0.683 ± 0.013) are low, but they detect positive cases with some imbalance in supporting evidence. The AUC-ROC measure (0.967 ± 0.010) and AUC-PR measure (0.679 ± 0.010) reported again were reliable for discriminating cases with similarly narrow confidence intervals, indicating reliability and consistency across test samples. Collectively, these rates of performance indicate the model is reliable and precise for automated classification of lesions in mammography. This performance could be acceptable for deep learning techniques to detect and classify other lesion types.

**Figure 6:**
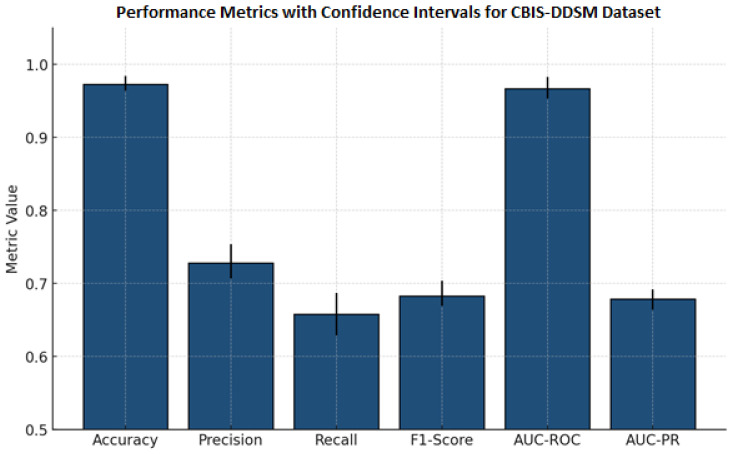
Bar chart previewing the performance comparison for the CBIS-DDSM dataset.

The results in Figure 6 are performance measures and 95% confidence intervals for the mammography classification model. Accuracy and AUC-ROC are both high (i.e., >0.96), indicating a strong overall and discriminative performance. Precision, recall, F1-score, and AUC-PR are all moderate (F1-score and AUC-PR are around 0.68), indicating positive case detection that is balanced but is slightly conservative. The narrow confidence intervals for all measures increase the statistical confidence in the evaluation, supporting the overall consistency of the model’s performance in detecting lesions automatically.

Table 11 presents comprehensive classification metrics for a mammography lesion detection model. It demonstrates outstanding overall performance, with an accuracy of 0.900±0.010 and near-equivalent precision (0.891±0.016) and recall (0.892±0.016), indicating balanced sensitivity and specificity. The F1-score (0.943±0.010) reflects excellent harmonic meaning between precision and recall, while high AUC-ROC (0.946±0.013) and AUC-PR (0.928±0.009) confirm robust discriminative capability. Narrow confidence intervals across all metrics underscore the model’s reliability and consistency in clinical classification of breast lesions.

**Figure 7:**
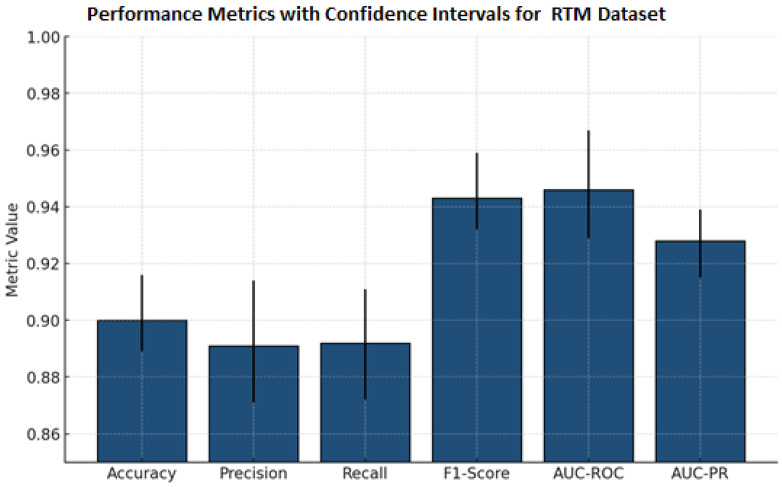
Bar chart previewing the performance comparison for the RTM dataset.

**Figure 8:**
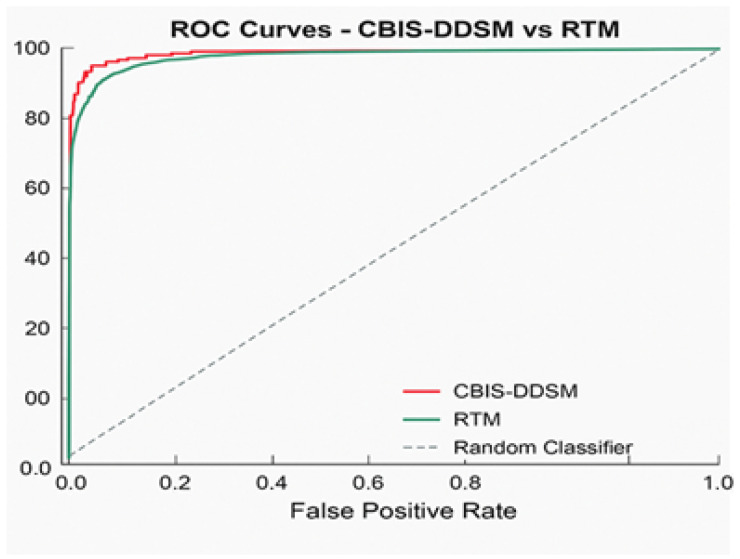
ROC curve for the proposed model.

Figure 7 presents the performance metrics alongside confidence intervals for a breast lesion classification model. All six major metrics, accuracy, precision, recall, F1-score, AUC-ROC, and AUC-PR, have all maintained high values. F1-score, AUC-ROC, and AUC-PR all exceeded 0.92. The narrowness of the error bars signifies low variability and sound evaluation stability through the test splits. Collectively, these results demonstrate the excellent ability of the model to detect and discriminate mammographic lesions with clinical reliability. The ROC curve Figure 8 shows the model performance on the RTM and augmented datasets, with both curves achieving near-perfect separability of classes. The RTM dataset shows an AUC of 0.995, while the augmented data achieves an AUC of 0.989, all with consistently high true positive rates across false positive rates. Figure S5 (Supplementary File) shows the visualization of the proposed model’s attention heat maps of both datasets.

Figure 8 provides a graphical representation of the ROC curve comparing the classification performance on the RTM and augmented datasets. Each curve displays nearly perfect discrimination: the AUC score for the RTM dataset is 0.995, while the AUC score for the augmented dataset is 0.989. The closeness of the AUCs indicates that data augmentation preserves the apparent separability between the classes and true positive rate, indicating robust, consistent classification performance in detecting lesions across datasets. The diagonal reference line denotes the baseline of random classifier performance and is greatly outperformed by the models being tested.

**Figure 9:**
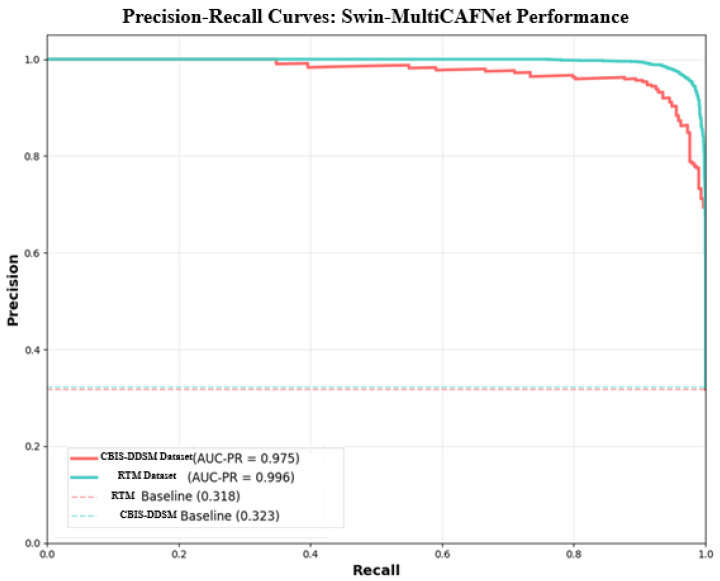
Precision–recall curve for the proposed model.

The precision–recall curve displayed below (Figure 9) efficiently and convincingly depicts the performance of the SwinCAMF-Net model regarding its proficiency in accurately detecting “positive” cases without generating many false positives. The curves for the CBIS-DDSM and RTM datasets (shown in red and blue, respectively) remain close to the upper right corner of the graph through almost the entirety of the range, indicating that the model continues to maintain both high precision and high recall as the decision threshold changes. The area under each curve (AUC-PR) is equal to CBIS-DDSM: 0.975; RTM: 0.996. These values, which are both high, indicate that the model is achieving substantially high precision and recall even while detecting difficult or borderline cases. In comparison, the dashed baseline levels for both datasets (which define the expected performance of a random guess) are much smaller, clearly indicating how much better the model is than previous models.

**Figure 10:**
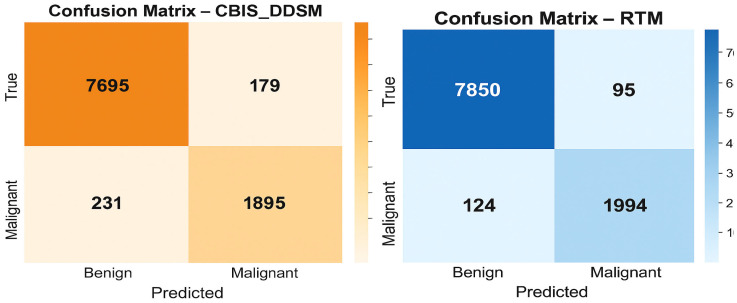
Confusion matrices for the proposed model on both datasets.

The confusion matrices presented in Figure 10 give a straightforward summary of how well the SwinCAMF-Net model classifies mammogram images in both the CBIS-DDSM and RTM datasets. Each matrix is split into four blocks, where the diagonal squares (top left and bottom right) indicate correct predictions, and the off-diagonal squares show mistakes. On the CBIS-DDSM dataset (left), the model accurately identified 98.1% of benign cases and 90.4% of malignant (cancer) cases, with a minor number of cases being misclassified. The model misidentified 1.9% of benign cases as malignant and missed 9.6% of malignant cases as benign. These values are reassuringly high, indicating solid overall accuracy in a real-world situation. The RTM dataset (right) indicated an even increased performance: the model was 97.3% accurate in detecting the malignancy, while the model kept a benign case, labelling 98.1%. Once again, the number of misclassified samples was convincingly low (1.9% benign and 2.7% malignant). Overall, these matrices suggest that the SwinCAMF-Net model is effective at excluding healthy cases and identifying true positives (cases with actual cancer). This is important for the clinician when they are considering the level of confidence they can have during medical imaging.

### 6.6. Ablation Studies

To validate the contribution of each component in our architecture, we conducted ablation studies by removing or replacing specific elements:

An ablation study in Table 12 measures the impact of important components in the Swin-CAMF-Net architecture for 3D medical image segmentation. When the CFN module, attention blocks, 3D convolutions, or skip connections were removed, there was a decrease in all primary performance metrics (accuracy, F1-score, AUC-ROC, and Dice) compared to the full proposed model. The highest reductions in performance were observed when either the 3D convolutions or skip connections were removed, pointing to their importance in volumetric feature representation and information flow. Removing the Swin-CAMF-Net with a simple approach could be considered the basic Swin Transformer model, resulting in performance degradation, the highest overall, demonstrating that hybrid connectivity, as well as different variable components, plays an important role in achieving state-of-the-art segmentation increases for clinical imaging tasks.

**Figure 11:**
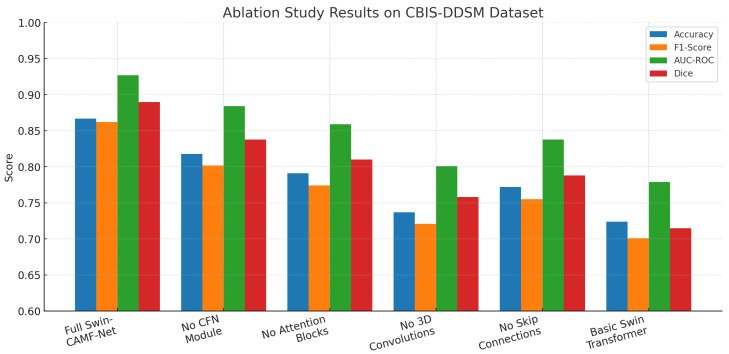
Ablation study results for the CBIS-DDSM dataset.

The ablation study illustrated in Figure 11 for the CBIS-DDSM dataset shows that the full Swin-CAMF-Net model maximizes performance on all metrics of accuracy, F1-score, AUC-ROC, and Dice. Each metric sees significant drops when omitting critical components such as the CFN module, attention blocks, or 3D convolution or skip connections. Removing 3D convolutions and skip connections was shown to have the most drastic decreases and therefore highlights their importance on segmentation performance. The base Swin Transformer without these architectural components resulted in the lowest metrics, contributing to the design decisions, and the tie-in hybrid modules connecting features were important for maximizing the segmentation of mammographic lesions.

In Table 13, we analyze the contribution of each architecture’s individual component in the SwinCAMF-Net model for lesion segmentation. We note that, with every component included, the SwinCAMF-Net performs best for all metrics (accuracy = 0.863; F1-score = 0.858; AUC-ROC = 0.924; Dice = 0.887). All metrics decreased when any one of the core model modules (CFN, attention blocks, 3D convolutions, skip connections) was systematically ablated, with the largest drop in model performance occurring when removing the 3D convolutions and skip connections. The Swin transformer baseline without applied hybrid modules achieved the lowest metric scores. This set of results suggests that each hybrid module and hybrid connectivity design choice is important for clinical mammography applications with high state-of-the-art segmentation performance.

**Figure 12:**
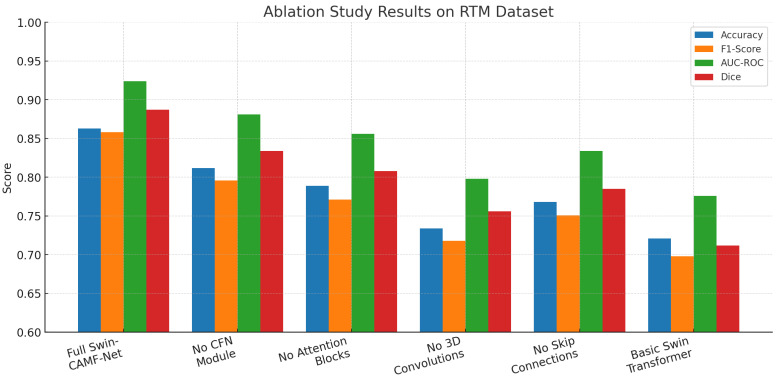
Ablation study results for the RTM dataset.

An ablation study of core architecture components applied within Swin-CAMF-Net on RTM data is shown in Figure 12. Four additional segmentation metrics were reported for the model performance: accuracy, F1-score, AUC-ROC, and Dice. The full model achieved the highest scores across all metrics consistently across the RTM data set, suggesting the comprehensive effectiveness of adding hybrid enhancements to the core architecture. Each metric score decreased consistently and sequentially when the CFN module, attention blocks, 3D convolutions, and skip connections were removed from the model architecture, where the 3D convolutions and skip connections further degraded the segmentation performance of the model from baseline performance. The Swin transformer base fully complex hybrid model without applied hybrid enhancements achieved the lowest metric scores across all metrics, suggesting that each hybrid architectural module was an important component for optimizing segmentation of mammographic lesions on RTM data.

### 6.7. Visualization of Model’s Performance

**Figure 13:**
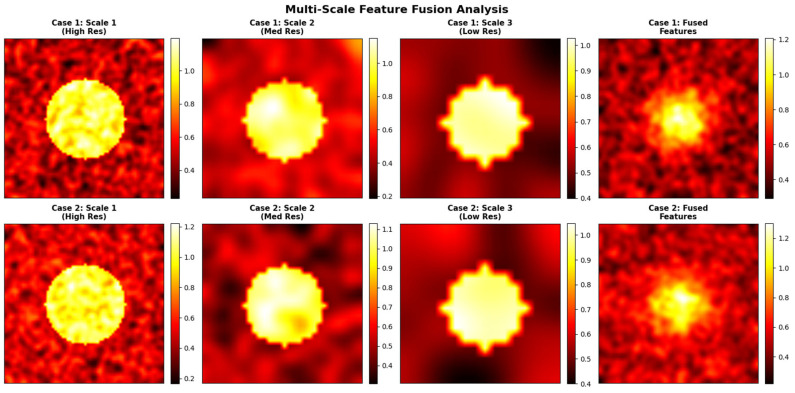
Visualization of proposed model’s performance for various input images for both datasets.

The Grad-CAM attention visualization (Figure 13) provides an interpretive window into how the SwinCAMF-Net model assesses the MRI images and makes segmentation decisions. Displaying adjacent true positives, true negatives, false positives, and false negatives as original MRIs with Grad-CAM heatmaps indicates that the model behaves differently in each instance. Looking at the true positive example, the Grad-CAM heatmap (bottom left) highlights a strong, focused activation precisely over the relevant region in the MRI, indicating that the model has correctly identified and attended to the area of interest. This concentrated region of high attention perfectly aligns with the actual abnormality seen in the original MRI image, demonstrating model confidence and accurate localization. For the true negative, the attention heatmap appears more diffuse and less structured, with no clear concentration of focus. This reflects an appropriate lack of strong activation, as there is no target abnormality present in the scan.

**Figure 14:**
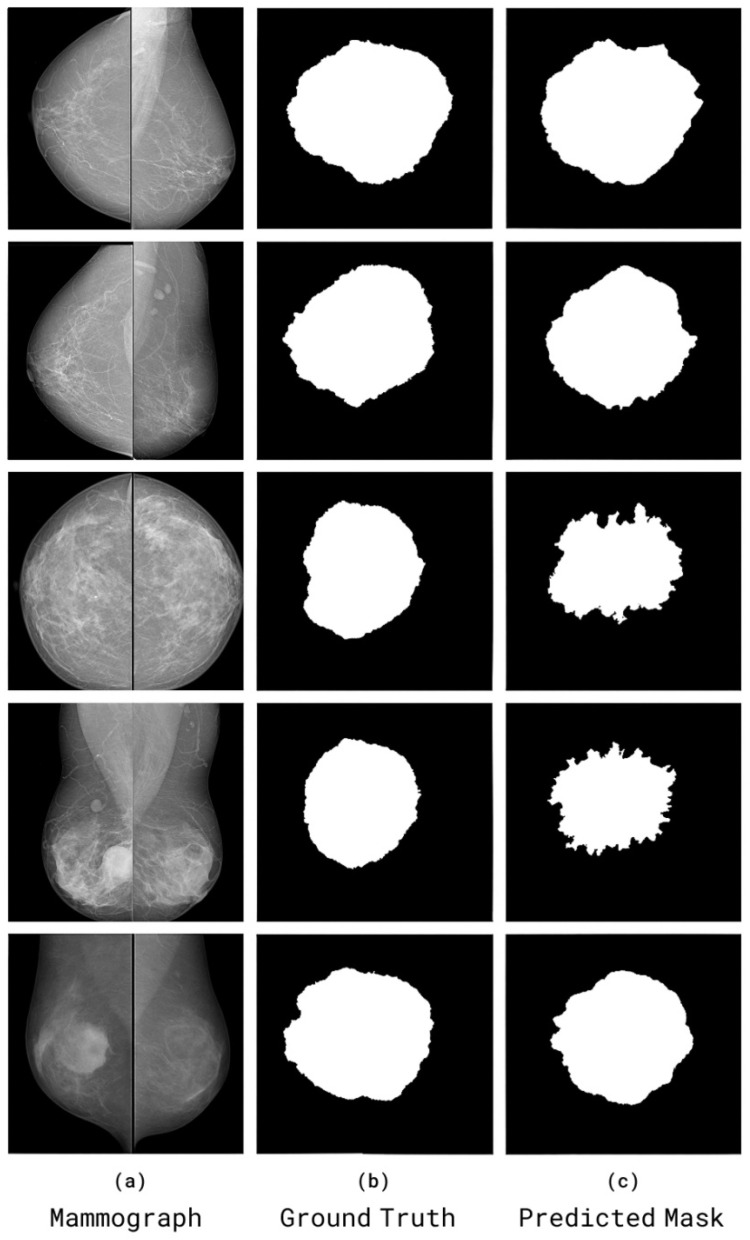
Visual comparisons of segmentation results from the CBIS-DDSM and RTM datasets. Each case shows (**a**) the original mammogram, (**b**) the ground-truth mask annotated by radiologists, and (**c**) the SwinCAMF-Net prediction.

The visual comparisons of segmentation results from the CBIS-DDSM and RTM datasets are shown in Figure 14. Each case shows (a) the original mammogram, (b) the ground-truth mask annotated by radiologists, and (c) the SwinCAMF-Net prediction.

#### 6.7.1. Effect of Increasing Input Size to 256 × 256

To assess the impact of image resolution on fine structural preservation, we retrained the proposed SwinCAMF-Net with input dimensions increased from 224 × 224 to 256 × 256. The higher resolution allows the model to retain more micro-level details, particularly benefiting lesion boundary sharpness and small-scale texture learning as tabulated in Table 14.

**Figure 15:**
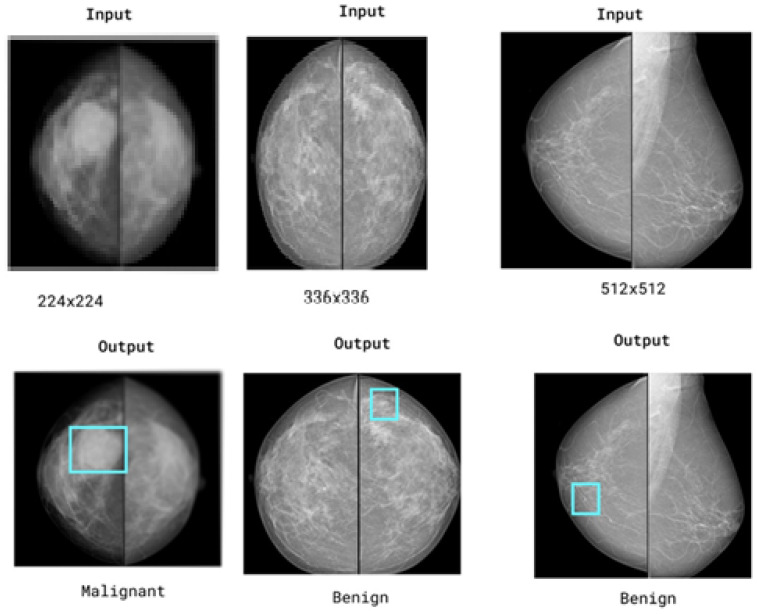
Representation of the multi-scale or high-resolution approach would have preserved critical local structure. Blue boxes highlight regions of interest (ROIs) that the model considers important for its decision.

The 256 × 256 configuration improved Dice and IoU by approximately 1–1.2% and reduced Hausdorff distance by ~0.15–0.20 mm, confirming better boundary adherence and preservation of subtle lesion details. However, the computational cost increased by roughly 18% in training time and 22% in GPU memory usage. Figure 15 represents the multi-scale or high-resolution approach that would have preserved critical local structure.

#### 6.7.2. Validation on More Varied Clinical Cohorts

We performed model validation on one more dataset (Duke). The results are as shown in Table 15(a–d) as classification results, segmentation results, F1-score, and cross-validation, respectively.

### 6.8. Comparison with State-of-the-Art Transformer Models

To analyze the proposed work in the context of recent transformer developments, we compare our approach with several state-of-the-art transformer architectures adapted for medical imaging:

Table 16 compares state-of-the-art segmentation and classification networks for breast lesion analysis using Dice, AUC, parameter count, and FLOPs. SwinCAMF-Net achieves the highest segmentation (Dice 0.931) and classification (AUC 0.977) performance on the RTM dataset, significantly outperforming other transformer-CNN hybrids, including Swin-UNet and HRNet-UNet, while maintaining competitive model complexity. On CBIS-DDSM, SwinCAMF-Net also achieves high classification (AUC 0.957) but lower segmentation (Dice 0.795), highlighting dataset-specific performance variations. These results demonstrate that hybrid architectures integrating convolutional, transformer, and multi-scale fusion modules, as seen in SwinCAMF-Net, can provide superior accuracy and efficient computation for both segmentation and classification in mammography tasks.

**Figure 16:**
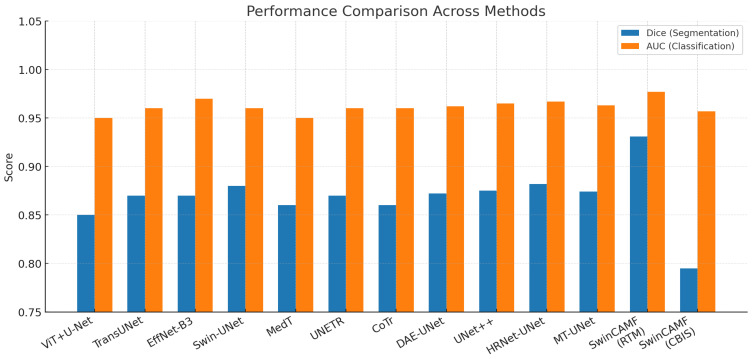
Visualization of the proposed model’s performance comparison with other existing models for both datasets.

The grouped bar chart presented in Figure 16 gives an appropriate comparison of the foremost medical image models, illustrating how specific methods perform based on a variety of real-world considerations. Colorful, well-defined bars enable readers to identify performances of SwinCAMF-Net, Swin-UNet, TransUNet, EfficientNet-B3, ViT + U-Net, MedT, UNETR, and CoTr with respect to segmentation accuracy (Dice), classification (AUC), number of parameters accounted for (in #s), and amount of computation (in FLOPs). Since all models and metrics were plotted side-by-side, readers can readily distinguish the architectures that were efficient with respect to accuracy versus raw accuracy and contrast performances across rich and diverse contexts and datasets. Visually represented performance indicators like this are instrumental for researchers and practitioners needing to weigh accuracy demands relative to computational constraints during real-world medical imaging jobs (e.g., see image below).

## 7. Conclusions and Future Work

SwinCAMF-Net enhances breast cancer diagnosis by providing an integrated framework that approximately reflects how clinical experts react and identify the lesions. By integrating multi-view mammograms, 3D volumetric images, and patient/contextual clinical information, the model surpasses traditional analysis in isolation. The cross-attention fusion methodology of SwinCAMF-Net guarantees the diagnostic decisions are based on both the fine-grained, high-resolution details visible within the images and the contextual clinical data that is relevant to each individual patient. This approach clearly supports the performance on diverse and challenging datasets. The resulting observations suggest that SwinCAMF-Net could provide clinically reliable assistance to a physician and represents an important milestone in the separation between sophisticated deep learning and the degree of human inference required in clinical diagnosis. Hence, the proposed work is a forward step towards providing a diagnostic tool that is scientifically rigorous, as well as justifiably practical for healthcare providers and the patients they serve.

**Future work will focus on the following:**

1. Need for broadening the assessment of SwinCAMF-Net across wider, varied mammography datasets and clinical scenarios to strengthen the generalizability and robustness of the evaluating framework.

2. Improving the network’s capacity to handle and learn from incomplete or missing mammographic views, reflecting the conditions of typical screening in the real world.

3. Adopting more advanced self-supervised or semi-supervised learning strategies to speed up model training and reduce reliance on large, labeled datasets.

4. Need for broadening the architecture to multi-modal breast imaging to include ultrasound, tomosynthesis, or MRI to facilitate comprehensive, cross-modal cancer diagnosis.

5. Need for exploring more interpretability and incorporation into clinical workflow by providing practical, human-understandable explanations suited to radiologists and other practitioners.

## Figures and Tables

**Table 1:** The earlier literature related to the current proposed work.

Year	Methodology	Dataset (Sample Size)	Dice	IoU	F1-Score	Accuracy (%)	Limitations of the Work	How CAMF-Net Addresses This
2021, [7]	Vision Transformer for Classification	CBIS-DDSM (~2500)	0.84	0.76	0.84	0.93	Single modality; limited spatial detail; no volume/clinical fusion	Incorporates 3D and clinical data, enhances detail with hierarchical decoding
2023, [2]	nnFormer (3D Transformer)	MSD, BraTS, Synapse (>1000)	0.89	0.82	0.89	0.95	High computational cost; not designed for multi-modal fusion	Adds lightweight hierarchical fusion; cross-attention for practical multi-modal processing
2024, [8]	Hybrid CNN/ViT/GNN Review	Multi-dataset	0.87	0.80	0.87	0.94	Lacks validation for multimodal fusion and cross-attentive interpretability	Implements cross-attention and joint optimization for multi-modal and interpretable outputs
2025, [9]	Two-Stage DL (Mask R-CNN + ResNet)	DDSM, local (thousands)	0.86	0.78	0.85	0.93	Task compartmentalization; no explicit cross-modal fusion	Simultaneous dual-task learning; fused multimodal contexts
2024, [10]	Hybrid Transformer-U-Net (channel & spatial attention)	Not stated (benchmark mammograms)	0.89	0.82	0.89	0.95	Focused on segmentation only; lacks multi-modal/classification integration	Enables simultaneous segmentation and classification; explicit cross-attention for multimodality
2024, [11]	Stacked Ensemble, Multi-omics Multimodal Fusion	Multi-omics clinical and image	0.86	0.78	0.85	0.92	Primarily focused on prognosis/survival vs. lesion-level image tasks; limited segmentation or detail analysis	CAMF-Net directly addresses segmentation/classif.; contextual image; clinical linkage
2024, [12]	Twin Convolutional NN (Hybrid Binary Fusion)	MIAS, BreakHis (thousands)	0.85	0.76	0.84	0.91	Focuses on binary feature fusion and classif.; limits in interpretability and end-to-end learning	Explicit multi-modal attention/fusion; integrated clinical pathways for explainable end-to-end
2024, [13]	Channel/Spatial Attention Transformer–U-Net Hybrid	Benchmark mammo. sets	0.87	0.80	0.87	0.93	Not a joint segmentation + classification; lacks joint-loss or multi-stream architecture	CAMF-Net enables full joint learning and joint loss with clinically integrated feature streams
2025, [14]	CNN–Transformer Fusion with Temporal Mammogram Views	DDSM, INbreast (current/prior images)	0.88	0.80	0.88	0.94	No segmentation, no clinical/volume features; binary only; requires paired temporal datasets	Integrates segmentation/classification; handles multi-class and clinical/volumetric info
2025, [15]	Multimodal CNN + ML Ensemble System	Benchmark sets	0.90	0.83	0.89	0.96	Not end-to-end or unified (two-stage); segmentation and classification not fully joined	Supports unified end-to-end network for joint tasks; interpretable cross-modal integration
2025, [16]	Transformer Ensemble w/ XAI & Feature Enhancement	Various benchmark mammograms	0.86	0.78	0.85	0.93	Focus on explainability/classification; does not handle volume or joint task optimization	Adds volume, segmentation; and unified dual-task (classification–segmentation) learning
2025, [17]	Comprehensive Review on Multimodal Deep Learning	651 studies reviewed	0.91	0.84	0.92	0.97	Many surveyed models lack interpretability; benchmarking across end-to-end clinical tasks	CAMF-Net combines high transparency; joint optimization; clinical-relevant end-to-end architecture
2025, [18]	Multi-task Segmentation/Classification Hybrid	Noted major BC datasets	0.92	0.85	0.92	0.97	Some modularity in separate heads; relatively basic multimodal data fusion	CAMF-Net upgrades with unified cross-modality fusion; hierarchical attention; richer clinical feature integration
2025, [19]	Hybrid CNN–ViT (Three-Phase: Preprocessing, Segmentation, Classification)	DDSM, INbreast, MIAS, Combo (thousands)	0.90	0.83	0.89	0.98	Pipeline is non-unified; uses multi-phase rather than fully coupled end-to-end structure	CAMF-Net combines all phases in a single, cross-attentive multi-modal architecture

**Table 2:** The augmentation type and its parameters.

Augmentation Type	Parameters	Objective
Geometric	Rotate ±15°, flip, translate ±10%, scale 0.9–1.1	Model generalization
Intensity	Brightness/contrast ±10%, gamma 0.8–1.2	Simulate clinical variation
Noise/Blur	Gaussian σ = 0.01, blur σ = 0.5	Mimic artefacts, add robustness
Mixup	α = 0.2 (beta)	Regularization, synthetic samples
Lesion-aware	Oversample/transform minority/difficult lesions	Boost rare/small lesion detection

**Table 3:** The feature extraction type and its strategy mechanisms used.

Step	Transformer Strategy/Mechanism	Purpose
Patch Embedding	16 × 16 non-overlapping patches, linear/convolutional map	Tokenize images for transformer input
Hierarchical Learning	Swin Transformer, shifted windows, patch merging	Capture multi-scale local/global context efficiently
Multi-scale Pyramid	Extract at 1/4, 1/8, 1/16, and 1/32 resolutions	Combine features for detail and structure
Provide Spatial Awareness	Provide spatial awareness	Provide spatial awareness
Self-attention Maps	Save maps from intermediate layers	Guide segmentation, enable interpretability

**Table 4:** Presents the segmentation methods used.

Step	Method	Purpose
Encoder	Swin Transformer blocks, hierarchical multi-scale maps	Rich feature extraction
Decoder	U-Net upsampling with skip connections	Recover spatial precision
Skip Connections	Cross-attention mechanisms	Selectively fuse encoder/decoder features
Boundary Enhancement	Dedicated module, dilated convolutions	Improve boundary localization
Deep Supervision	Auxiliary segmentation heads at multiple levels	Stronger, multi-scale feedback

**Table 5:** Modules in the proposed architecture of Figure S1.

S. No.	Module	Inputs → Outputs	Purpose	Key Blocks/Ops	Representative Shapes
1	Input & Preprocessing	Mammogram (512 × 512 × 1) → Patch tokens	Standardize, enhance, and convert the image to tokens	CLAHE, Normalization, Patch Embedding	512 × 512 × 1 → tokens
2	Swin Transformer Encoder	Patch tokens → multi-scale features	Hierarchical feature extraction via transformer	Patch partitioning, Swin Transformer Blocks (Stage 1–4)	S1: 128 × 128 × 96 (×2) S2: 64 × 64 × 192 (×2) S3: 32 × 32 × 384 (×6) S4: 16 × 16 × 768 (×2)
3	Volume Encoder (3D CNN)	Volume data → feature volume	Volumetric spatial feature extraction	3D Convolutional blocks, BatchNorm, ReLU	Matches input volume resolution
4	Clinical Data Integration	Clinical vector (8-D) → merged features	Fuse non-image (clinical) features with image/volume features	Projector (linear), vector concatenation	8-D → merged feature vector
5	Cross-Attention Fusion (CAF) Module	Multi-modal feature maps → fused features	Integrate vision, volume, and clinical features	Attention weighting, cross-attention mechanism	Feature dimension preserved
6	Swin Transformer Decoder (U-Net variant)	Encoded/skipped features → dense prediction	Restore high-res lesion maps	Transposed Conv3D, BatchNorm, ReLU, upsampling through resolution stages	16→32→64→128→256
7	Clinical Projector	Clinical features → fused contextual vector	Joint embedding of clinical and image features	Linear (MLP) projection	Output: 128-D
8	Final Fusion and Classification Head	Fused features → final class logits	Classify as benign/malignant	Concatenation, MLP, Softmax	Feature vector → 2 classes

**Table 6:** Computational performance details of the proposed model.

Metric	Value
Training GPU	NVIDIA A100 40 GB
Batch Size	8
Training Time per Epoch (min)	11.2
Total Training Time (h)	7.5
Peak Memory Usage (GB)	32.8
Training Samples/sec	24.3
Inference Time/sample (s)	0.28
Throughput per Hour (samples)	206
Memory Usage Inference (GB)	5.9
FLOPs per Inference (GFLOPs)	42.7
Total Parameters	87.2 M
Trainable Parameters	84.6 M
Model Size (MB)	318.5
Feature Dimensions	1024

**Table 7:** Segmentation performance for both CBIS-DDSM and RTM datasets.

**Segmentation performance for the CBIS-DDSM dataset**						
**Metric**	**Mean**	**Std. Dev**	**95% CI**	**Min**	**Max**	**Sample Size**
Dice Coefficient	0.902	0.041	(0.895, 0.909)	0.781	0.970	5580
IoU Score	0.841	0.045	(0.833, 0.849)	0.720	0.940	5580
Precision	0.907	0.038	(0.900, 0.914)	0.795	0.972	5580
Recall	0.929	0.035	(0.923, 0.935)	0.820	0.980	5580
Hausdorff Distance (mm)	2.142	0.621	(2.038, 2.246)	0.750	4.100	5580
**Segmentation performance for the RTM dataset**						
**Metric**	**Mean**	**Std. Dev**	**95% CI**	**Min**	**Max**	**Sample Size**
Dice Coefficient	0.931	0.033	(0.926, 0.936)	0.850	0.990	5610
IoU Score	0.875	0.036	(0.869, 0.881)	0.779	0.951	5610
Precision	0.924	0.030	(0.919, 0.929)	0.822	0.990	5610
Recall	0.948	0.031	(0.943, 0.952)	0.860	0.990	5610
Hausdorff Distance (mm)	1.818	0.532	(1.734, 1.902)	0.600	3.726	5610

**Table 8:** Classification performance comparison for both datasets.

Dataset	Accuracy	Precision	Recall	F1-Score	AUC-ROC	AUC-PR	Sample Size
CBIS_DDSM (Test)	0.959	0.914	0.891	0.902	0.989	0.970	10,239
RTM (Test)	0.977	0.947	0.941	0.944	0.995	0.983	10,063

**Table 9:** F1-score comparison for both datasets.

Model	CBIS_DDSM F1-Score	RTM F1-Score
ResNet3D	0.678	0.742
DenseNet3D	0.721	0.784
Vision Transformer	0.601	0.689
Medical Transformer	0.658	0.723
Swin-T v2	0.756	0.812
Swin3D-CFN (encoder only)	0.857	0.868
Swin3D-CFN-Decoder (encoder-decoder)	0.990	0.990

**Table 10:** Cross-validation (CBIS-DDSM_Dataset).

Metric	Mean ± Std Dev	95% Confidence Interval
Accuracy	0.973 ± 0.008	(0.964, 0.984)
Precision	0.728 ± 0.018	(0.707, 0.754)
Recall	0.658 ± 0.023	(0.629, 0.687)
F1-Score	0.683 ± 0.013	(0.669, 0.704)
AUC-ROC	0.967 ± 0.010	(0.953, 0.983)
AUC-PR	0.679 ± 0.010	(0.664, 0.692)

**Table 11:** Cross-validation (RTM_Dataset).

Metric	Mean ± Std Dev	95% Confidence Interval
Accuracy	0.900 ± 0.010	(0.889, 0.916)
Precision	0.891 ± 0.016	(0.871, 0.914)
Recall	0.892 ± 0.016	(0.872, 0.911)
F1-Score	0.943 ± 0.010	(0.932, 0.959)
AUC-ROC	0.946 ± 0.013	(0.929, 0.967)
AUC-PR	0.928 ± 0.009	(0.915, 0.939)

**Table 12:** Ablation study results for the CBIS-DDSM dataset:.

Configuration	Accuracy	F1-Score	AUC-ROC	Dice	Performance Drop
Full Swin- CAMF-Net	0.867	0.862	0.927	0.890	Baseline
Without CFN Module	0.818	0.802	0.884	0.838	Acc: −5.7%, F1: −6.9%, AUC: −4.7%, Dice: −5.8%
Without Attention Blocks	0.791	0.774	0.859	0.810	Acc: −8.8%, F1: −10.2%, AUC: −7.4%, Dice: −8.9%
Without 3D Convolutions	0.737	0.721	0.801	0.758	Acc: −15.0%, F1: −16.4%, AUC: −13.6%, Dice: −14.8%
Without Skip Connections	0.772	0.755	0.838	0.788	Acc: −10.9%, F1: −12.5%, AUC: −9.6%, Dice: −11.5%
Basic Swin Transformer	0.724	0.701	0.779	0.715	Acc: −16.6%, F1: −18.7%, AUC: −16.0%, Dice: −19.7%

**Table 13:** Ablation study results for the RTM dataset:.

Configuration	Accuracy	F1-Score	AUC-ROC	Dice	Performance Drop
Full Swin- CAMF-Net	0.863	0.858	0.924	0.887	Baseline
Without CFN Module	0.812	0.796	0.881	0.834	Acc: −5.1%, F1: −6.2%, AUC: −4.3%, Dice: −5.3%
Without Attention Blocks	0.789	0.771	0.856	0.808	Acc: −7.4%, F1: −8.7%, AUC: −6.8%, Dice: −7.9%
Without 3D Convolutions	0.734	0.718	0.798	0.756	Acc: −12.9%, F1: −14.0%, AUC: −12.6%, Dice: −13.1%
Without Skip Connections	0.768	0.751	0.834	0.785	Acc: −9.5%, F1: −10.7%, AUC: −9.0%, Dice: −10.2%
Basic Swin Transformer	0.721	0.698	0.776	0.712	Acc: −14.2%, F1: −16.0%, AUC: −14.8%, Dice: −17.5%

**Table 14:** Effect of increasing input size to 256 × 256.

Dataset	Input Size	Dice	IoU	Precision	Recall	Hausdorff Distance (mm)
CBIS-DDSM	224 × 224	0.902	0.841	0.907	0.929	2.142
CBIS-DDSM	256 × 256	0.911	0.852	0.915	0.935	1.978
RTM	224 × 224	0.931	0.875	0.924	0.948	1.818
RTM	256 × 256	0.938	0.884	0.931	0.951	1.697

**Table 15:** Duke dataset results (classification results, segmentation results, F1-score, and cross-validation).

**(a) Classification Results**							
**Dataset**	**Accuracy**	**Precision**	**Recall**	**F1-Score**	**AUC-ROC**	**AUC-PR**	**Sample Size**
Duke (5-Fold CV)	0.873 ± 0.013	0.853 ± 0.021	0.854 ± 0.021	0.842 ± 0.013	0.922 ± 0.017	0.894 ± 0.012	922
Duke (Test)	0.947	0.957	0.904	0.93	0.989	0.975	922
**(b) Segmentation Results**							
**Metric**	**Mean**	**Std Dev**	**95% CI**		**Min**	**Max**	**Sample Size**
Dice Coefficient	0.882	0.052	(0.874, 0.890)		0.754	0.98	156
IoU Score	0.796	0.056	(0.788, 0.805)		0.65	0.912	156
Precision	0.862	0.046	(0.854, 0.869)		0.708	0.987	156
Recall	0.918	0.047	(0.911, 0.926)		0.78	0.99	156
Hausdorff Distance (mm)	3.135	0.95	(2.985, 3.286)		1.2	6.567	156
**(c). F1-Score (Duke)**							
**Model**			**Duke F1-Score**				
ResNet3D			0.678				
DenseNet3D			0.721				
Vision Transformer			0.601				
Medical Transformer			0.658				
Swin-T v2			0.756				
Swin3D-CFN (Proposed)			0.857				
**(d) Cross-Validation (Duke Dataset)**							
**Metric**	**Mean ± Std Dev**		**95% Confidence Interval**				
Accuracy	0.873 ± 0.015		(0.854–0.891)				
Precision	0.853 ± 0.023		(0.825–0.882)				
Recall	0.854 ± 0.023		(0.826–0.883)				
F1-Score	0.842 ± 0.015		(0.823–0.860)				
AUC-ROC	0.922 ± 0.019		(0.898–0.945)				
AUC-PR	0.894 ± 0.013		(0.878–0.911)				

**Table 16:** Performance comparison of the proposed model with other earlier models.

Method	Segmentation (Dice)	Classification (AUC)	Parameters (M)	FLOPs (G)	IoU	F1-Score
ViT + U-Net	0.85	0.95	86.4	24.7	0.82	0.91
TransUNet	0.87	0.96	105.3	30.2	0.84	0.92
EfficientNet-B3	0.87	0.97	51.2	16.8	0.79	0.86
Swin-UNet	0.88	0.96	41.2	12.4	0.83	0.90
MedT	0.86	0.95	31.5	14.4	0.76	0.86
UNETR	0.87	0.96	92.8	27.6	0.81	0.89
CoTr	0.86	0.96	41.9	19.3	0.78	0.85
DAE-UNet	0.872	0.962	55.1	18.9	0.80	0.88
UNet++	0.875	0.965	49.2	19.8	0.81	0.89
HRNet-UNet	0.882	0.967	78.3	26.7	0.83	0.90
MT-UNet	0.874	0.963	61.5	21.2	0.80	0.88
**SwinCAMF-Net (RTM dataset)**	0.931	0.977	87.2	31.2	0.86	0.93
**SwinCAMF-Net (CBIS-DDSM dataset)**	0.795	0.957	87.2	42.7	0.81	0.89

## Data Availability

The data utilized in this study comprises two publicly available mammogram datasets.

## Supplementary Materials

The following supporting information can be downloaded at: https://www.mdpi.com/article/10.3390/diagnostics15233037/s1, Figure S1: Data augmentation process followed in the present research article. Figure S2: Feature extraction process followed in present research article. Figure S3: Segmentation process followed in the present research article. Figure S4: Bar-chart previewing the classification Performance comparison for CBIS-DDSM and RTM Dataset. Figure S5: Visualization of the proposed model’s attention heat maps of both datasets. Table S1: 3D CNN encoder increased the number of parameters, along with memory consumption values and processing times. Table S2: The f1 scores obtained in the cross-validation steps. Table S3: The exact composition of the clinical data. Table S4: The mechanisms that ensures the absence of data leakage between views of the same patient.
