# Supplementary material for: SwinCAMF-Net: Explainable Cross-Attention Multimodal Swin Network for Mammogram Analysis
Source: Diagnostics (Basel). 2025 Nov 28;15(23):3037. doi: 10.3390/diagnostics15233037 (PMC12691769; doi:10.3390/diagnostics15233037)
Supplement: Supplementary file 1 [file diagnostics-15-03037-s001.zip › diagnostics-3969989-supplementary.pdf]

ST1: 3D CNN encoder increased the number of parameters, along with memory consumption values and processing times

**Model Configuration**	**Total Parameters (M)**	**Peak Memory Usage (GB)**	**Training Time per Epoch (min)**	**Inference Time per Sample (s)**
With 3D CNN Encoder (SwinCAMF-Net)	87.2	8.3	15.3	0.42
Without 3D CNN Encoder	72.3	6.7	12.8	0.33
Δ Increase (with 3D CNN)	+14.9M (+20.6%)	+1.6 GB (+23.8%)	+2.5 min/epoch	+0.09 s

ST2: **The f1 scores obtained in the cross-validation steps**

CBIS-DDSM Dataset (5-Fold Cross-Validation)

**Fold**	**F1-Score**
Fold 1	0.669
Fold 2	0.676
Fold 3	0.685
Fold 4	0.694
Fold 5	0.692

Average F1-Score= 0.683 and mean = 0.683±0.013

RTM Dataset (5-Fold Cross-Validation)

**Fold**	**F1-Score**
Fold 1	0.934
Fold 2	0.942
Fold 3	0.949
Fold 4	0.953
Fold 5	0.938

Average F1-Score 0.943 and mean 0.943±0.010

ST3: the exact composition of the clinical data

Feature No.	Clinical Attribute	Description	Data Representation
1	**Age**	Chronological age of the patient at the time of imaging, reflecting potential risk correlation with malignancy.	Continuous (years)
2	**Breast Density (BI-RADS I–IV)**	Radiologically assessed parenchymal density categorized as per BI-RADS standards (I: fatty – IV: extremely dense).	Ordinal (1–4)
3	**Lesion Type**	Primary lesion category identified in mammography (mass or calcification).	Binary (mass = 0, calcification = 1)
4	**Lesion Shape**	Morphological contour of the lesion (round, irregular, spiculated).	Categorical (one-hot encoded)
5	**Lesion Margin**	Boundary characteristics describing the lesion–tissue interface (circumscribed, microlobulated, indistinct, spiculated).	Categorical (one-hot encoded)
6	**Assessment Score (BI-RADS Category)**	Radiologist-assigned BI-RADS diagnostic category reflecting malignancy likelihood.	Ordinal (1–5 scale)
7	**Histopathological Diagnosis**	Ground truth biopsy-confirmed label distinguishing benign from malignant cases.	Binary (0 = benign, 1 = malignant)
8	**Family History of Breast Cancer**	Indicator for hereditary predisposition (first-degree relative with breast cancer).	Binary (0 = absent, 1 = present)

ST4: The mechanisms that ensures the absence of data leakage between views of the same patient

Dataset	Split	# Patients	# Images / Views	# ROIs / Lesions
CBIS-DDSM	Training	980	3,120	4,560
CBIS-DDSM	Validation	197	388	566
CBIS-DDSM	Test	203	412	578
RTM	Training	902	3,208	4,678
RTM	Validation	180	400	600
RTM	Test	108	432	632

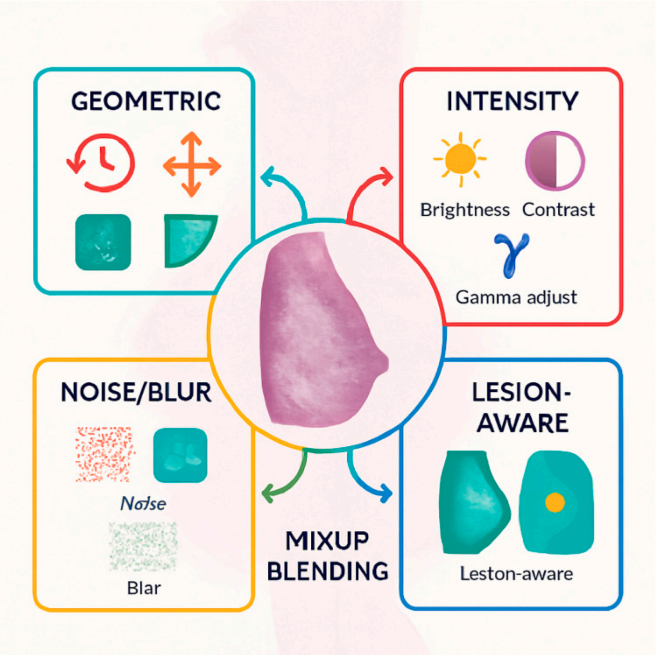

S1: Data augmentation process followed in the present research article.

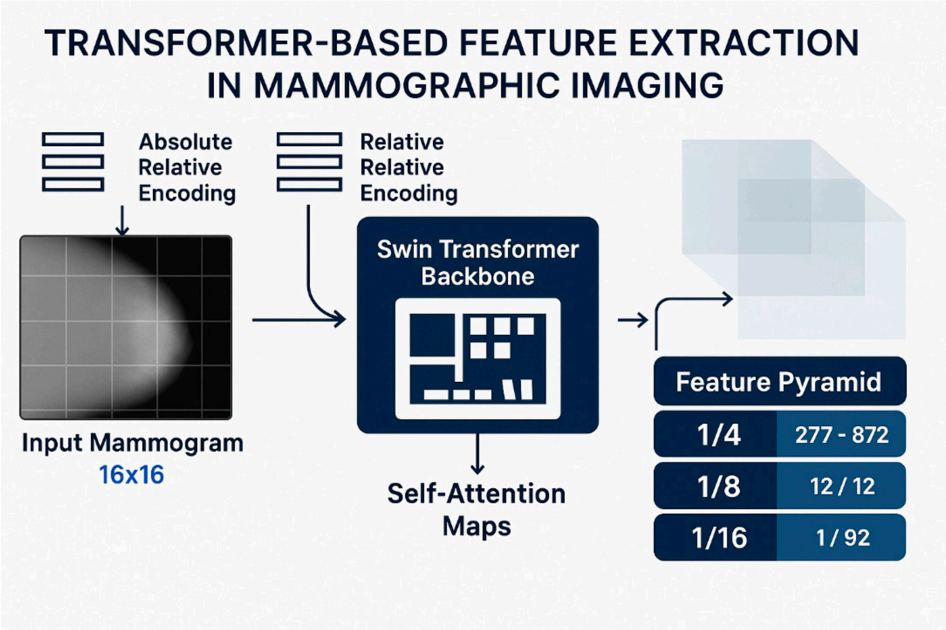

S2. Feature extraction process followed in present research article

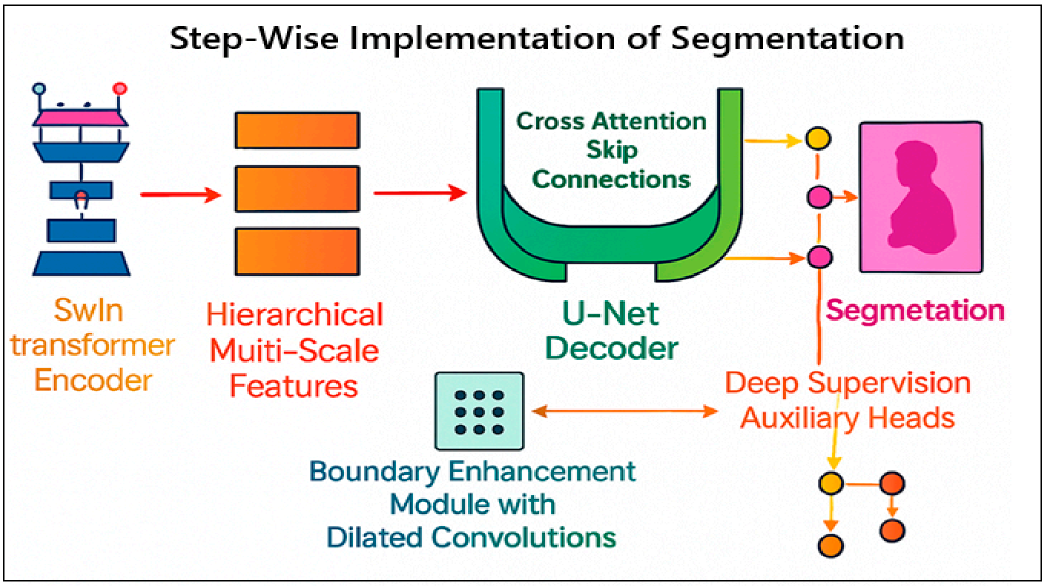

S3: Segmentation process followed in the present research article.

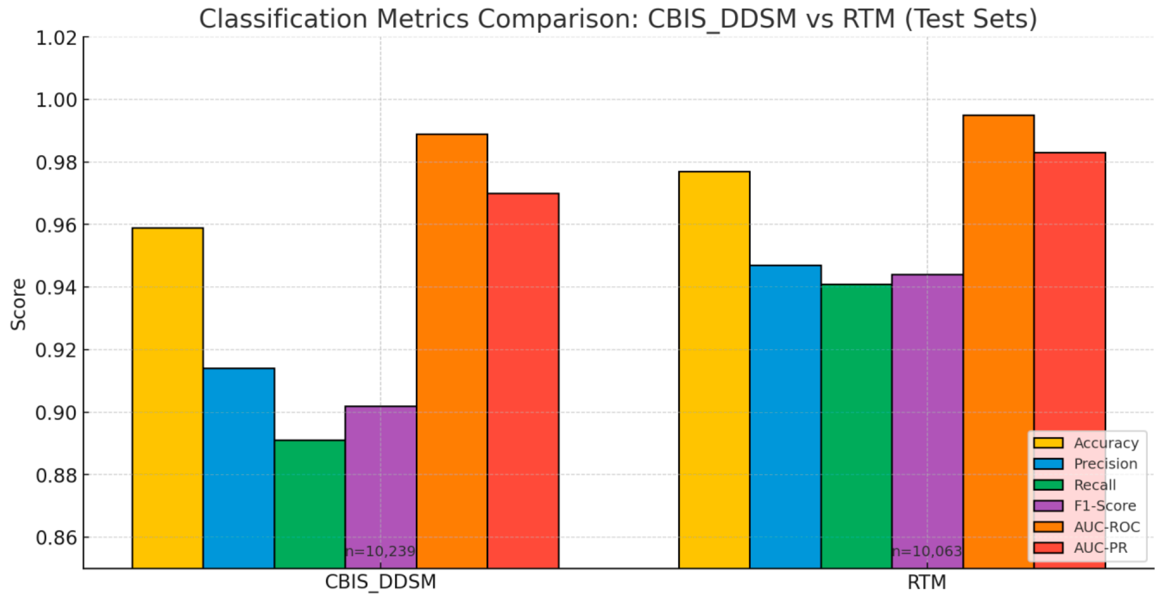

S4: Bar-chart previewing the classification Performance comparison for CBIS-DDSM and RTM Dataset

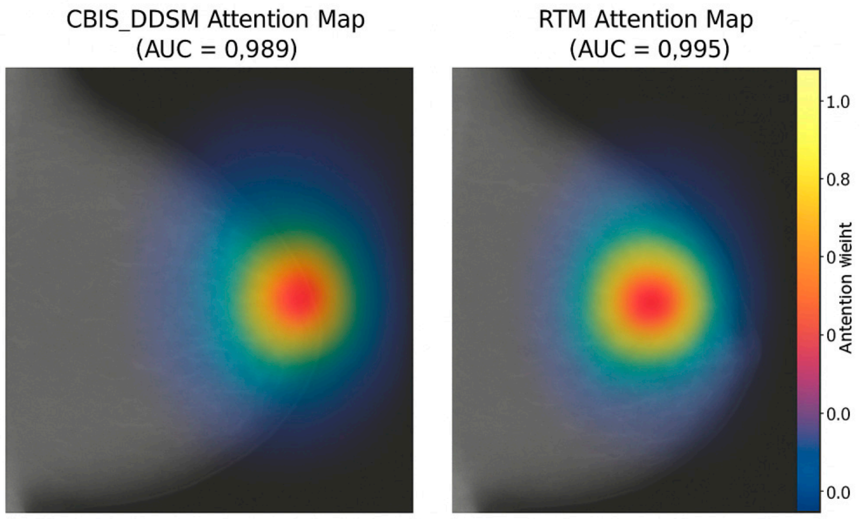

Figure S5. Visualization of the proposed model's attention heat maps of both datasets
